# University-industry collaboration: The impact of postdoctoral workstations on labor investment efficiency

**DOI:** 10.3389/fpsyg.2022.955935

**Published:** 2022-10-13

**Authors:** Yiding Liu, Kefu Yi, Guanhua Huang

**Affiliations:** ^1^Business School, University of International Business and Economics, Beijing, China; ^2^School of Economics and Management, Inner Mongolia University, Hohhot, China; ^3^Branch of Jiangxi, Agricultural Development Bank of China, Nanchang, China

**Keywords:** university-industry collaboration, postdoctoral workstation, labor investment efficiency, difference-in-differences, optimal labor level

## Abstract

This paper investigates whether managers use knowledge transferred from university-industry collaboration when making investment decisions on labor. To establish causality, we use a difference-in-difference method based on the staggered establishment of postdoctoral workstations in Chinese firms. We find that postdoctoral workstations enable managers to improve labor investment efficiency and thus help mitigate over- and under-investment problems in labor, and the higher the operational quality of the workstation, the more significant the increase in investment efficiency. This finding is robust to utilizing the event study approach, placebo test, propensity score matching, instrumental variable, and entropy balancing. Brain gain and knowledge transfer effects between universities and industries are two plausible mechanisms. Furthermore, the main effect is more pronounced for firms located closer to prestigious universities, firms are non-state-owned enterprises, human-capital-intensive, have political connections, and without national fellows’ lead. Our findings suggest that brain gain in firms does not merely increase or reduce labor investments *Per se*, but rather inspires managers to maintain optimal labor levels through knowledge transfer processes.

## Introduction

The impact of university-industry (hereafter, U-I) collaboration on firm value has been well-documented (Bastos et al., 2021). Through this collaboration, universities are the primary producers of knowledge, integrating teaching and research activities. Firms, in turn, play a role in putting knowledge to practical use. Viewed broadly, U-I collaboration includes more than just the partnership between the two parties. It also contributes to the benefits of continued encouraging the exchange of information, knowledge, and technology, among others, all of which are critical components for firms’ innovative activities (Cohen et al., 2002; de Wit-de Vries et al., 2018). In 1985, as one of its U-I collaboration policies, China implemented the industrial postdoctoral system to boost a specific collaborative pattern, in which firms and universities are involved together in a joint postdoctoral project (Stith et al., 2011). By 2018, there were 3,727 industrial postdoctoral research centers, known as ‘postdoctoral workstations’ established in eligible corporate entities. Firms in the high-tech industry (e.g., HUAWEI collaborated with Peking University and Tsinghua University) have priority in the process of government approval for postdoctoral workstations.

Prior studies focus primarily on postdoc individuals, including the influencing factors for their career intentions in industries and the challenges they face (Hayter and Parker, 2019). However, it is still a debate whether postdoctoral workstations benefit or deteriorate firms’ value-enhancing activities, relative to the other U-I collaboration patterns. The positive view argues that joint postdoctoral workstations act as nodes linking firms and universities, generating inclusive knowledge and therefore contributing to a firm’s competitive advantage (e.g., Salimi et al., 2016; Santos et al., 2020). For instance, while not specifically focused on postdoctoral workstations, Thune (2009) states: ‘for the firm, PhDs and post-doctorates represent a channel to absorb tacit knowledge. To this point, postdoctoral workstations would serve as a primary vessel of knowledge transfer in U-I collaboration, and thus facilitate the accumulation of human capital in firms. In contrast, some prior studies find that the industrial postdoctoral system may result in negative outcomes because of the misalignment of postdoctoral education and deficiency of skills required for employment in the industrial sector (Smaglik, 2014; Stenard and Sauermann, 2016). We are motivated to investigate labor investment efficiency (hereafter, LIE) because the effect of postdoctoral workstations on the firm’s human capital is largely overlooked.

The purpose of this paper is to investigate whether managers use knowledge transferred from U-I collaboration when making labor investment decisions. To address the endogeneity of U-I collaboration, we use the manually collected postdoctoral project data from the Ministry of Human Resources and Social Security (hereafter, MOHRSS) of China and exploit a quasi-natural experiment setting based on the staggered establishment of postdoctoral workstations in A-share listed firms. We find that the presence of postdoctoral workstations leads to a 1.89% reduction in inefficient labor investment, which corresponds to shrinking the gap between the expected optimal level by 11.5%.

Although China is not the only setting that can provide empirical evidence on this question, it is appealing to investigate the determinants of labor investment in an underdeveloped but strong government intervention context. Additionally, unlike the United States, which has the best research universities and increasingly concentrated talents from all over the world (MacLeod and Urquiola, 2021), China is experiencing a brain drain of high-end talents. Its policies designed to increase the higher educated population led to a boom in domestic PhD holders; however, the problems of how to retain these talents and promote their contribution to economic development still exist. The scarcity of scientific research talents, increasing labor costs, and poor labor investment efficiency are problems common to many emerging markets (Li et al., 2020; Wei et al., 2020). It is, therefore, both theoretically and practically important to investigate the impact of postdoctoral workstations on firms’ LIE in emerging countries. For this reason, our findings provide insights into the validity of joint postdoctoral workstations, and more generally, U-I collaboration, for emerging markets that face structural changes in their labor market.

Our work is related and contributes to the burgeoning literature on efficient labor investment. Previous literature focuses on a variety of corporate governance determinants of LIE based on information asymmetry and agency theory (see for example Ghaly et al., 2020; Jung et al., 2014; Kong et al., 2018; Sualihu et al., 2021), while neglecting to examine whether a firm’s human resource development strategy itself affects LIE. Our research stands as an initial attempt at this issue by investigating the economic implication of postdoctoral workstations for firms’ human resource development. Furthermore, by emphasizing brain gain and knowledge transfer effects as they matter for managerial employment decisions, we respond to the call of Hayter and Parker (2019) to investigate the postdoctoral tacit knowledge spillover phenomenon within the employment sector. Hence, our findings provide managers with a deeper understanding of the knowledge transfer role which U-I collaboration plays in influencing labor investment, thereby assisting them to make more informed employment decisions.

The paper is organized into five sections. The following section 2 develops the hypothesis. Section 3 describes the data and methodology. Section 4 presents and discusses the empirical results, and the final section concludes.

## Hypothesis development

Based on information asymmetry and agency theory, a growing amount of accounting and finance research has addressed the determinants of LIE. For instance, Jung et al. (2014) document that the quality of a firm’s financial reporting is positively correlated with LIE, suggesting that firms with high information transparency are also more effective in terms of labor investment. Ghaly et al. (2020) show that institutional investors with a long-term perspective reduce both labor over- and under-investment problems when they gain control of a company. Their finding is consistent with the argument that institutional investors will mitigate agency conflicts through monitoring effects to increase LIE. Among the related studies in the Chinese setting, Kong et al. (2018) and Luo et al. (2020) find that the promotion of local government officials and policy uncertainty leads to inefficient labor investment. In short, the existing literature on LIE is rare in terms of applying theories other than information asymmetry and misalignment in principle-agent incentives.

On the one hand, postdocs who are recruited and trained through U-I collaboration are regarded as a potential brain gain, and their presence may facilitate LIE in several ways. First, as documented in economic literature, innovation as a source of corporate competitive advantage is an amply studied topic. For example, Siegel et al. (2003) suggest that direct employment of postdoctoral researchers in U-I collaboration is an extremely efficient way of knowledge transfer and technology innovation, even if the short-term financial performance is not outstanding. Mansfield (1995) and Johnson (2018) highlight that an academic invention stemming from U-I collaboration is exploited to reap financial gain. That is, in the economic sense, postdoctoral innovation is achieved through the firm’s commercial transaction, i.e., commercialization (Shi et al., 2020). According to the capital-skills complementarity hypothesis (Duffy et al., 2004; Parro, 2013), the representation of skilled labor brings unique experienced ideas to put knowledge into practical use and resolve the issues related to the development of commercialization. Consequently, the role of skilled labor in U-I collaboration is more effective when compared to unskilled labor. Building on this conjecture, managers are likely to reduce under-investment in labor since they need to hire more skilled labor with experienced learning capabilities, focus on the management of tacit knowledge, to turn the postdocs’ innovative ideas into practical use for commercialization (Hanisch et al., 2009).

Second, as the high costs incurred in recruiting and maintaining postdocs will impose temporary budgetary constraints on human resource development, it will force managers to take action to cut back on over-investment. Moreover, since postdoctoral researchers in U-I collaboration are more aware of the frontiers of industrial advancement (Salimi et al., 2016), they can advise managers on human resource development, therefore resulting in a considerable reduction in the risk associated with labor investments. Last, being permitted to establish a postdoctoral workstation means that firms can train postdocs internally under their corporate strategy, making it more flexible to adjust their labor investments in comparison to external recruiting. Meanwhile, Plewa et al. (2014) propose that firms can hire graduates who have been educated to meet their firm-specific competencies directly from universities through U-I collaboration. Therefore, postdoctoral workstations enable firms to lower their search and recruit costs for high-end employees and thus win the “talent war” (Cao and Rees, 2020) in the labor market. These arguments suggest that the postdoctoral workstation under U-I collaboration should improve a firm’s LIE, the following hypothesis is proposed:

*Hypothesis 1*: The postdoctoral workstation has a positive impact on LIE.

On the other hand, there are several reasons why the presence of postdoctoral workstations may not positively affect the efficiency of the labor investment process. First, the postdoctoral workstation may undermine LIE because there is an “educational mismatch” phenomenon—that is, a situation where postdocs’ scientific research ability does not fully meet the actual needs of firms—that may make it difficult for managers to capitalize on the knowledge transferred from U-I collaboration (Stenard and Sauermann, 2016). In a similar vein, according to Hayter and Parker (2019), although postdocs are knowledgeable about scientific concepts and research methods, they still lack practical experience and do not understand how to effectively transform scientific research ideas into business opportunities. Second, Lin and Chiu (2016) argue that industrial postdoctoral positions are ‘refuge-seeking jobs’ for the less talented PhD holders who fail to find an academic position. In particular, as higher education expands alongside a significant shrinking in academic positions, the demand for people with a PhD is reaching saturation point (Smaglik, 2014). Employment pressure pushes PhD holders with insufficient ability to choose non-academic postdoctoral positions as a temporary transition, creating a market for ‘lemons’ with adverse selection, which results in inefficient labor investment. Finally, as the postdoctoral appointment is a temporary position by nature (e.g., postdoctoral contracts in China are typically for 2 years), numerous barriers to effective knowledge transfer would occur due to the lack of long-term incentives. The above viewpoint shows that firms need to pay much attention to the operation quality of postdoctoral workstations to effectively play the role of human resources and promote the efficiency of labor investment.

*Hypothesis 2*: The higher the operation quality of postdoctoral workstations in firms, the more obvious the effect of improving the efficiency of LIE.

In addition, China has a special political environment and talent cultivation system. For sensitive political factors, we introduce the variable of whether or not there is a connection with the Ministry of Science and Technology (hereafter, MOST) (*Postdoc_MOST*); relationship capital inadvertently influences the boundaries of resource barriers and is a reflection of a firm’s competitive advantage in an open environmental system (Bao and Peng, 2015). Siegel et al. (2003) found that the political resources of firms significantly improved knowledge transfer and absorption, achieving higher levels of innovation performance by compensating for the lack of firms’ R & D capabilities, reflecting an increase in LIE. Likewise, we further distinguish between workstations led by national fellows and those without (*Postdoc_Fellows*). As the highest level of domestic scientific research, fellows of the Chinese Academy of Sciences and the Academy of Engineering have a non-negligible contribution to technological innovation (Fisman et al., 2018). Therefore, it is usually considered a critical factor in the workstation of U-I collaboration, which is to improve the efficiency of industry-university-research cooperation, enrich innovation resources, and accelerate the transformation of research results into economic benefits (Li et al., 2018), may create incentives for postdocs in the lemon market to transfer their knowledge more effectively, leading to more opportunities for commercialization and more efficient corporate labor investment.

*Hypothesis 3a*: Postdoctoral workstations with political connections with MOST have more significant improvements in LIE.

*Hypothesis 3b*: Postdoctoral workstations led by national fellows have a more pronounced improvement in LIE.

## Empirical design

### Sample and data sources

We obtain data to test our conjecture from two sources. First, we start with a comprehensive list of Chinese A-share listed firms with fundamental financial data retrieved from the China Stock Market and Accounting Research (CSMAR) database. Second, to form a list of firms that are allowed for recruiting and training postdocs, we manually collect information on the joint postdoctoral project on the websites of the MOHRSS. After excluding firms in the financial industry and observations with incomplete data, the final sample consists of an unbalanced panel of 10,392 firm-year observations from 2,457 firms during the period 2011 to 2019. To mitigate the effect of outliers, all continuous variables are winsorized at the 1st and the 99th%levels except for firm age.

### Measure of labor investment efficiency

We use a two-step procedure to measure LIE (Jung et al., 2014; Sualihu et al., 2021). In the first step, we estimate the expected net hiring following the economic fundamentals model developed by Pinnuck and Lillis (2007) in Equation (1). Then, we calculate the deviation of actual net hiring from expected net hiring and construct the abnormal net hiring. Specifically, we denote the absolute value of residuals obtained from Equation (1) as labor investment inefficiency (*|AbnNetHire|*).


(1) $$\begin{array}{l} NetHiring_{i,t}=\beta_{0}+\beta_{1}Return_{i,t}\\ +\beta_{2}MVRank_{i,t-1}\\ +\beta_{3}ROA_{i,t}+\beta_{4}Qratio_{i,t-1}\\ +\beta_{5}LtDebt_{i,t-1}+\overset{1}{\underset{j=0}{\sum }}\theta_{j}SaleG_{i,t-j}\\ +\overset{1}{\underset{j=0}{\sum }}\varphi_{j}\Delta ROA_{i,t-j}+\overset{1}{\underset{j=0}{\sum }}\rho_{j}\Delta Qratio_{i,t-j}\\ +\overset{5}{\underset{j=0}{\sum }}\tau_{j}LossBin_{i,t-1,j}+Industry\;FE+\varepsilon_{i,t}, \end{array}$$

where, the subscripts *i* and *t* refer to the firm *i* and the year *t*, respectively. *NetHiring* represents the actual net hiring, which is the percentage change in the number of employees. *Return* is the total annual stock return; *MVRank* is the logarithmic value of market value at the beginning of the year, ranked into percentiles; *ROA* is the return on assets; *Qratio* is the ratio of current assets minus inventory to current liabilities; *LtDebt* is the ratio of long-term debt to total assets; and *SaleG* is the percentage change in sales revenue. We also add the current-year (prior-year) change in *ROA*, which is Δ*ROA_t_* (*ΔROA*_*t*–1_). In a similar method, we construct the *ΔQratio*. *LossBin* is five dummy variables indicating each interval of prior-year *ROA* of length 0.005 from 0 to −0.025. The model also includes China Securities Regulatory Commission (hereafter, CSRC) 2-digit industry fixed effects to control for unobserved industry characteristics affecting net hiring.

### Model specification

When firms are permitted to recruit postdocs by MOHRSS, they begin to benefit from the potential brain gain and knowledge transfer through U-I collaboration. Thus, the permitting events provide a quasi-natural experiment setting to employ the multi-period Difference-in-Difference (hereafter, DiD) design in a staggered manner. To test the relationship between postdoctoral workstation and LIE, we estimate the following regression model:


(2) $$\begin{array}{l} {\left|AbnNetHire\right|}_{i,t}=\beta_{0}+\beta_{1}Postdoc_{i,t}\\ +\gamma CV_{i,t-1}+\mu_{i}+\lambda_{t}+\varepsilon_{i,t}, \end{array}$$

where, the dependent variable is labor investment inefficiency (*|AbnNetHire|*), which is an inverse measure of LIE. The variable of interest, *Postdoc*, is a dummy variable with a value of one if the firm establishes the joint postdoctoral workstation collaborated with universities and zero otherwise. A negative coefficient on *Postdoc* (*β*_1_) represents that the postdoctoral workstation increases LIE, and vice versa. ***CV*** denotes a vector of control variables that may be associated with labor investment, including firm size (*Insize*), firm leverage ratio (*Lev*), market-to-book ratio (*MB*), cash flow (*Cfo*), firm age (*Age*), operating profit (*Profit*), return on equity (*ROE*), ownership concentration (*Top*1), dividend policy (*DivDum*), net hiring volatility (*Vol_Nethiring*), and revenue volatility (*Vol_Revt*). Since firm investment is a decision that reflects previous managerial expectations, all the control variables are 1 year lagged. To mitigate endogeneity concerns caused by unobservable firm-specific factors and omitted variables, we include the firm- (*μ_i_*) and year- (*λ_t_*) fixed effects. Finally, we cluster standard errors at the firm level. Definitions of key variables are shown in Table 1.

**Table 1:** Variable definitions.

Variables	Definition
*|AbnNetHire|*	Labor investment efficiency (inverse measure), the absolute values of the residuals from Equation (1)
*Postdoc*	Postdoctoral workstation, an indicator equal to one if the firm establishes the joint postdoctoral workstation collaborated with universities, and zero otherwise
*Insize*	Firm size, the natural logarithm of total assets
*Lev*	Firm leverage, the ratio of total debt to total assets
*MB*	Market-to-book ratio, the ratio of the market value to the book value of total assets
*Cfo*	Cash flow, the ratio of operating cash flows to total assets
*Age*	Firm age, calculated as the current year minus the establishment year
*Profit*	Operating profit, calculated as profit from operation divided by revenue
*ROE*	Return on equity, calculated as net income divided by book value of equity
*Top1*	Ownership concentration, the fraction of shares held by the largest shareholder
*DivDum*	Dividend payment, an indicator equal to one if the firm paid dividends in the previous year, and zero otherwise
*Vol_Nethiring*	Hiring volatility, calculated as the five-year rolling-window standard deviation of the change in the number of employees
*Vol_Revt*	Revenue volatility, calculated as a five-year rolling-window standard deviation of revenue
*Other_invest*	Abnormal other non-labor investments, the absolute values of the residuals from the non-labor investments model as defined in Sualihu et al. (2021)

## Empirical results and discussion

### Descriptive statistics and correlation matrix

Table 2 presents descriptive statistics for variables used in our primary tests. The average (median) of LIE is equal to 0.165 (0.095) with a standard deviation of 0.274. These statistics are similar to Ding et al. (2021) results with an average (median) equal to 0.151 (0.084) with a standard deviation of 0.216, who also use Chinese listed firms’ data to calculate LIE. Our primary variable of interest, *Postdoc*, has a mean of 0.329 and a standard deviation of 0.470, which is in line with Quan et al. (2020) findings. The statistics of postdoctoral workstation measures show that, on average, approximately 32.9% of our firm-year observations have a partnership with a university in the form of joint postdoctoral workstations. Other control variable descriptive statistics largely agree with Jung et al. (2014) and Luo et al. (2020).

**Table 2:** Summary statistics for key variables.

Variables	*N*	Mean	SD	P25	Median	P75
*|AbnNetHire|*	10,392	0.165	0.274	0.044	0.095	0.175
*Postdoc*	10,392	0.329	0.470	0	0	1
*Insize*	10,392	22.401	1.264	21.511	22.196	23.084
*Lev*	10,392	0.444	0.196	0.291	0.439	0.588
*MB*	10,392	0.611	0.246	0.417	0.605	0.798
*Cfo*	10,392	0.047	0.064	0.010	0.045	0.085
*Age*	10,392	16.763	5.386	13	17	20
*Profit*	10,392	0.056	0.160	0.016	0.059	0.123
*ROE*	10,392	0.059	0.137	0.024	0.065	0.117
*Top1*	10,392	0.337	0.145	0.224	0.315	0.429
*DivDum*	10,392	0.788	0.409	1	1	1
*Vol_Nethiring*	10,392	0.112	0.231	0.017	0.040	0.097
*Vol_Revt*	10,392	0.216	0.563	0.020	0.047	0.137
*Other_invest*	10,392	0.044	0.040	0.019	0.036	0.055

This table presents summary statistics for key variables in this study. The sample comprises 10,392 observations from 2,457 firms during the period 2011 to 2019.

Next, we conduct the Pearson’s pairwise correlation analysis in Table 3. The Pearson’s correlation coefficient of *|AbnNetHire|* and *Postdoc* is −0.043 and significant at 1%. Other control variable descriptive statistics largely agree with Jung et al. (2014) and Luo et al. (2020). In addition, we calculate the Variance Inflation Factor (VIF) scores in the primary test. The mean VIF score is 1.71, and the VIF scores for all variables are lower than 4. Furthermore, we observe that the majority of the control variables are significantly associated at the low to medium level, which suggests that the potential multi-collinearity is not a serious concern.

**Table 3:** Pearson correlation.

Variables	(1)	(2)	(3)	(4)	(5)	(6)	(7)	(8)	(9)	(10)	(11)	(12)	(13)	(14)
(1) *|AbnNetHire|*	1.000													
(2) *Postdoc*	**−0.043**	1.000												
(3) *Insize*	0.005	**0.065**	1.000											
(4) *Lev*	0.003	0.014	**0.475**	1.000										
(5) *MB*	−0.025	0.007	**0.569**	**0.422**	1.000									
(6) *Cfo*	0.002	−0.002	**0.084**	**−0.183**	**−0.104**	1.000								
(7) *Age*	**−0.050**	**0.067**	**0.146**	**0.111**	**0.116**	**0.037**	1.000							
(8) *Profit*	**0.034**	0.009	**0.076**	**−0.347**	**−0.140**	**0.317**	**−0.035**	1.000						
(9) *ROE*	**0.070**	0.021	**0.135**	**−0.209**	**−0.132**	**0.317**	**−0.028**	**0.739**	1.000					
(10) *Top1*	**0.033**	**−0.127**	**0.258**	**0.085**	**0.125**	**0.111**	**−0.089**	**0.098**	**0.115**	1.000				
(11) *DivDum*	**−0.038**	**0.042**	**0.099**	**−0.147**	**−0.028**	**0.133**	**−0.026**	**0.243**	**0.238**	**0.056**	1.000			
(12) *Vol_Nethiring*	**0.142**	0.018	**0.563**	**0.240**	**0.239**	**0.076**	0.013	0.018	**0.130**	**0.166**	**0.044**	1.000		
(13) *Vol_Revt*	0.024	−0.012	**0.652**	**0.287**	**0.330**	**0.059**	0.013	0.007	**0.118**	**0.241**	**0.057**	**0.672**	1.000	
(14) *Other_invest*	**0.118**	−0.014	−0.022	**0.087**	**−0.028**	−0.011	**−0.090**	−0.003	**0.032**	0.024	**−0.044**	**0.041**	−0.023	1.000

This table presents Pearson pairwise correlation coefficients for key variables in this study. Values highlighted in boldface indicate significance at the 1% level.

### Basic regression results

#### The effect of postdoctoral workstations on LIE

Table 4 presents the major results regarding postdoctoral workstations and LIE. Column (1) shows the results of our full model, where we find that *Postdoc* is negative and significant at the 1% level, suggesting that brain gain into firms following the postdoctoral workstation establishment can reduce inefficient labor investment. In addition, non-labor investments (e.g., capital investments in physical assets such as property, plants, and equipment) are frequently made at the same time as labor investments, potentially resulting in LIE changes occurring simultaneously. Hence, managers’ decisions on labor investment are influenced by other non-labor investment efficiencies (Jung et al., 2014; Luo et al., 2020). To account for this effect, in Column (2), we further control for corporate physical capital investment efficiency (*Other_invest*) in our baseline specification. The coefficient continues to be statistically significant and has a real economic significance, that is, the establishment of postdoctoral workstation results in an increase of 11.5% (= 0.0189/0.165) in LIE relative to the sample mean.

**Table 4:** The effect of postdoctoral workstation on LIE.

Variables	Dependent Variable = *|AbnNetHire|*				
	Overall (1)	Overall (2)	Over-invest (3)	Under-invest (4)	PSM (5)
*Postdoc*	−0.0195*** (−3.393)	−0.0189*** (−3.310)	−0.0274** (−2.060)	−0.0101*** (−2.783)	−0.0146** (−2.277)
*Insize*	−0.0148*** (−3.532)	−0.0144*** (−3.525)	−0.0307*** (−2.943)	−0.0073*** (−2.948)	−0.0160*** (−3.471)
*Lev*	0.0060 (0.329)	−0.0103 (−0.569)	−0.0141 (−0.352)	0.0072 (0.520)	−0.0166 (−0.768)
*MB*	0.0238 (1.433)	0.0297* (1.779)	0.1306*** (3.213)	−0.0402*** (−3.968)	0.0350* (1.809)
*Cfo*	−0.0560 (−1.165)	−0.0503 (−1.046)	−0.0228 (−0.193)	0.0017 (0.048)	−0.0674 (−1.177)
*Age*	−0.0013** (−2.127)	−0.0011* (−1.717)	−0.0013 (−0.923)	−0.0007* (−1.705)	−0.0014** (−1.991)
*Profit*	−0.0176 (−0.694)	−0.0132 (−0.523)	−0.0465 (−1.044)	−0.0037 (−0.172)	0.0088 (0.275)
*ROE*	0.1688*** (4.613)	0.1559*** (4.358)	0.2607*** (4.017)	0.0990*** (3.470)	0.1406*** (3.539)
*Top1*	0.0400* (1.672)	0.0396* (1.666)	0.0906 (1.611)	0.0254* (1.778)	0.0397 (1.517)
*DivDum*	−0.0327*** (−4.070)	−0.0310*** (−3.904)	−0.0441** (−2.449)	−0.0209*** (−4.032)	−0.0281*** (−3.030)
*Vol_Nethiring*	0.2903*** (10.859)	0.2811*** (10.727)	0.6709*** (9.760)	0.0600*** (4.902)	0.2474*** (8.610)
*Vol_Revt*	−0.0565*** (−5.885)	−0.0534*** (−5.635)	−0.1338*** (−5.201)	−0.0076* (−1.720)	−0.0440*** (−4.237)
*Other_invest*		0.6695*** (5.582)	0.9283*** (4.266)	0.2001*** (2.854)	0.5385*** (3.735)
*Constant*	0.4941*** (5.893)	0.4538*** (5.608)	0.7814*** (3.826)	0.3148*** (6.297)	0.4976*** (5.565)
*Firm and Year*	Yes	Yes	Yes	Yes	Yes
*Adj*. *R^2^*	0.054	0.063	0.124	0.044	0.058
*N*	10,392	10,392	3,600	6,792	7298

This table reports the results for Equation (2). Columns (1)–(2) report the results with different control variables. Columns (3)–(4) report the results in the over-investment and under-investment sub-sample, respectively. Robust standard errors clustered at the firm level to derive t-statistics in parentheses. ***, **, and * represent significance at the 1, 5, and 10% levels, respectively.

In the spirit of Sualihu et al. (2021), we split the LIE into sub-samples according to employment decisions, and define positive (negative) abnormal net hiring with a positive (negative) sign on the residuals from Equation (1) as over-investment (under-investment). These results are reported in columns (3) and (4) of Table 4, respectively. The coefficients on *Postdoc* are still significantly negative, indicating that postdoctoral workstations can reduce both over- and under-investment at the same time.

In Table 5, we further decompose over-investment into over-hiring and under-firing by considering whether a firm’s labor force should grow or contract following the firm-specific economic fundamentals as Jung et al. (2014) defined. Specifically, a firm is over-hiring (under-firing) if it over-invests in labor when its expected level of net hiring is positive (negative) based on Equation (1). Likewise, we decompose under-investment into under-hiring (i.e., the expected level of net hiring is positive) and over-firing (i.e., the expected level of net hiring is negative) sub-samples. We find that each form of under-investment is mitigated as the coefficient on *Postdoc* is significantly negative; however, in columns (1), the results for over-hiring become insignificant. Collectively, we find that most specific types of LIE are facilitated by the presence of postdoctoral workstations. The above results indicate that the establishment of postdoctoral workstations by firms has a positive impact on LIE, supporting hypothesis H1.

**Table 5:** The effect of postdoctoral workstation on specific types of LIE.

**Variables**	Dependent Variable = *|AbnNetHire|*			
	Over-investment		Under-investment	
	Over-hiring (1)	Under-firing (2)	Under-hiring (3)	Over-firing (4)
*Postdoc*	−0.0214 (−1.396)	−0.0545** (−2.057)	−0.0093** (−2.323)	−0.0116* (−1.868)
*Insize*	−0.0335** (−2.548)	0.0383* (1.790)	−0.0096*** (−3.322)	−0.0126** (−2.246)
*Lev*	−0.0670 (−1.215)	−0.0257 (−0.420)	−0.0037 (−0.210)	0.0275 (1.430)
*MB*	0.1740*** (3.233)	−0.1799** (−2.143)	−0.0215* (−1.694)	−0.0416** (−1.999)
*Cfo*	−0.2086 (−1.457)	0.3555 (1.605)	−0.0096 (−0.218)	−0.0489 (−1.058)
*Age*	−0.0020 (−1.306)	−0.0037 (−1.309)	−0.0004 (−0.942)	−0.0013** (−2.109)
*Profit*	−0.0530 (−0.422)	−0.0446 (−0.730)	−0.0202 (−0.528)	−0.0149 (−0.683)
*ROE*	0.3989** (2.105)	0.3327*** (4.011)	0.1723*** (2.968)	−0.0023 (−0.100)
*Top1*	0.0651 (1.019)	−0.0070 (−0.069)	0.0337** (2.123)	−0.0102 (−0.437)
*DivDum*	−0.0526** (−2.352)	−0.0010 (−0.041)	−0.0323*** (−5.021)	0.0040 (0.661)
*Vol_Nethiring*	0.4742*** (6.990)	1.5173*** (6.614)	0.0521*** (3.984)	0.1401*** (3.940)
*Vol_Revt*	−0.1044*** (−3.696)	−0.2904*** (−3.879)	−0.0066 (−1.418)	−0.0022 (−0.165)
*Other_invest*	0.5935*** (2.900)	2.7163*** (4.331)	0.2254*** (2.938)	0.1295 (1.099)
*Constant*	0.8763*** (3.461)	−0.5775 (−1.373)	0.3613*** (6.335)	0.3977*** (3.571)
*Firm and Year*	Yes	Yes	Yes	Yes
*Adj*. *R^2^*	0.093	0.274	0.046	0.123
*N*	2,363	1,237	5,826	966

This table reports the results for Equation (2) on four specific types of abnormal net hiring (Over-hiring, Under-firing, Under-hiring and Over-firing) as Jung et al. (2014) defined. Robust standard errors clustered at the firm level to derive t-statistics in parentheses. ***, **, and * represent significance at the 1, 5, and 10% levels, respectively.

#### The effect of the quality of postdoctoral workstations on LIE

According to the “Opinions of the General Office of the State Council of the People’s Republic of China on Reforming and Improving the Postdoctoral System” ([2015] No. 87),^1^ the MOHRSS of the People’s Republic of China conducted a comprehensive evaluation every 5 years, including four aspects of workstation basic construction, recruitment and selection, work supervision, and achievements assessment. In particular, it will examine the “effectiveness of postdoctoral in transforming achievements” and “awards of postdoctoral projects.” Due to the high authority and comprehensiveness of the evaluation results, it is an ideal indicator to measure the quality of the operation of postdoctoral workstations (Huang et al., 2021). Moreover, Germain-Alamartine et al. (2020) pointed to the company’s collaborating university as a way of effectively screening postdoctoral candidates. In China, first-class universities are listed as “Double First Class,” “985 Project,” and “211 Project,” or affiliated with the Chinese Academy of Sciences, so we also use the level of partner institutions as a proxy. Operational quality of the workstations. Therefore, we used the workstation valuation level (*Postdoc_Eva*) and the collaborating university level (*Postdoc_Uni*) to measure the quality of workstation operations.

In Table 6, the coefficient of the independent variable is significant and negative at 1% regardless of whether it is the workstation evaluation level or the collaborating university level. As a result, the higher the quality of a firm’s postdoctoral workstation operation (better external evaluation results and higher level of the collaborating university), the faster the transfer of knowledge between industry-university-research teams and the lower the inefficiency of the enterprise’s labor investment. This finding provides support for Hypothesis 2.

**Table 6:** The effect of the quality of postdoctoral workstation on LIE.

Variables	Dependent Variable = *|AbnNetHire|*					
	Valuation level (1)	University level (2)	MOST(3)	Fellows (4)	Postdoc (5)	PSM (6)
*PDRC_Eva*	−0.024*** (−3.64)					
*PDRC_Uni*		−0.001*** (−2.85)				
*Postdoc_MOST*			−0.0269*** (−2.668)			
*Postdoc_Other*			−0.0192*** (−3.260)			
*Postdoc_Fellows*				−0.0076 (−0.660)		
*Postdoc_non-Fellows*				−0.0202*** (−3.401)		
*Iniv*					−0.001*** (−2.85)	
*Postdoc*						−4.220*** (−3.16)
*Constant*	0.465** (2.55)	0.229 (1.19)	0.4507*** (5.567)	0.4539*** (5.611)	−4.921*** (−6.44)	−1.934* (−1.83)
*Other Controls*	Yes	Yes	Yes	Yes	Yes	Yes
*Firm and Year*	Yes	Yes	Yes	Yes	Yes	Yes
*Adj*. *R^2^*	0.063	0.039	0.063	0.063	0.043	0.645
*N*	1,916	973	10392	10392	7298	7298

This table reports the results of the quality of postdoctoral workstations [columns (1)–(2)], workstations with political connections with MOST [columns (3)], workstations led by national fellows [columns (4)], and instrumental variable [columns (5)–(6)]. Robust standard errors clustered at the firm level to derive t-statistics in parentheses. ***, **, and * represent significance at the 1, 5, and 10% levels, respectively.

#### Robustness checks

##### Parallel trend assumption

**Figure 1:**
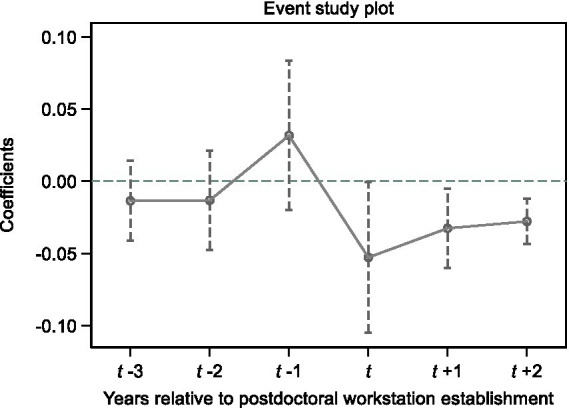
Parallel trend. This figure reports the annual dynamics of postdoctoral workstations on labor investment efficiency. Specifically, the figure plots the coefficients on *Postdoc* and the corresponding 95% confidence interval based on the event study approach.

We applied three additional processes to assess the robustness of our DiD design. The first is the parallel trend assumption, which asserts that there should be no difference between the treated and control firms before the treatment year. To that end, we conduct an event study approach in Figure 1 to examine the parallel trends. Specifically, *t* < 0 stands for the difference of the dependent variable (*|AbnNetHire|*) in the period before a firm establishes the postdoctoral workstation and *t* > 0 corresponds to the annual dynamic impact effect. In Figure 1, when *t* is less than 0, the 95% confidence interval for all three coefficients on *Postdoc* includes zero, indicating that the trends for LIE are indistinguishable for at least 3 years before the workstation establishment. By comparison, when *t* is greater than 0, the coefficients are negatively significant, demonstrating that firm-level brain gain has a long-term and persistent influence on the LIE.

##### Placebo test

**Figure 2:**
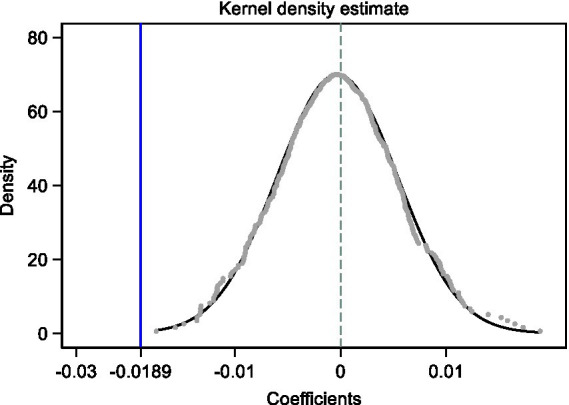
Placebo test. This figure reports the density of placebo coefficients on *Postdoc* from the 500 times assigning the postdoctoral workstation to firms randomly. The vertical blue line presents the benchmark coefficient on *Postdoc* reported in Column (2) of Table 4.

Our second process to check for robustness is the placebo test using a false treatment group. Following recent studies (Liu and Mao, 2019; Ding et al., 2021), we implement a non-parametric permutation test by randomly assigning the establishment of workstations to firms. We obtain 500 placebo coefficients on *Postdoc* after repeating this procedure 500 times to boost the test identifying power. Figure 2 demonstrates that the distribution of placebo coefficients is normally centered around zero with a tiny standard deviation, implying that the randomly assigned false treatment has no discernible effect. Furthermore, the benchmark coefficient in column (2) of Table 4, as indicated by the vertical reference line with an equivalent value of −0.0189, is located outside the lower tail of the distribution. These results support that the impact of postdoctoral workstations on LIE is robust rather than random.

##### Propensity score matching

Since the choice of whether to recruit and train postdocs is not random, potential biases may be related to firm characteristics. We choose all control variables related to basic firm characteristics are selected as covariates, using no replacement, one-to-one nearest neighbor propensity score matching (hereafter, PSM) method to match the control group to the treatment group. Ultimately, we constructed a PSM sample using 7,298 observations from 2,585 listed firms. In the PSM sample (see column (5) of Table 4), the postdoc coefficient is significantly negative, has a statistical significance level of less than 5%, and has a reduced coefficient value than the full sample regression, indicating that the full sample overestimates the impact of postdoctoral workstations on LIE, thus justifying the use of the PSM sample.

**Figure 3:**
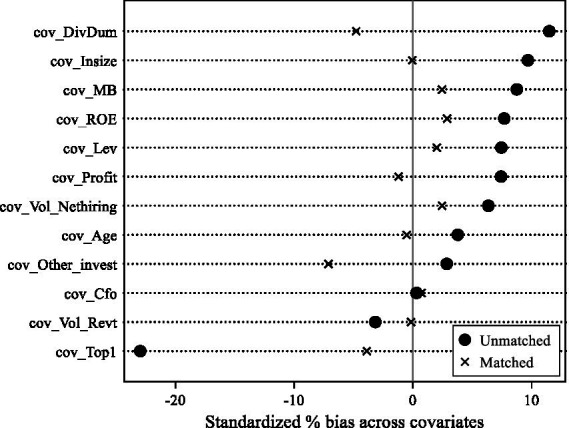
Balance Test. This figure reports the test for the proximity of treatment and control group covariate means after matching.

To verify whether the matching results eliminate systematic differences between the treatment and control groups, a balance test is required. From the test results in Figure 3, it can be seen that the standardized biases of the variables narrowed after matching. Specifically, in the unpaired samples, there was a significant difference in the means of covariates between the treatment and control groups, while in the paired sample groups, there was no significant difference in the means of covariates between the treatment and control groups, and standardized bias all within 10%. It indicates that the PSM sample satisfies the balance test, and the matching result is more satisfactory, which can correct the estimation bias caused by the “selection bias” of the sample.

**Figure 4:**
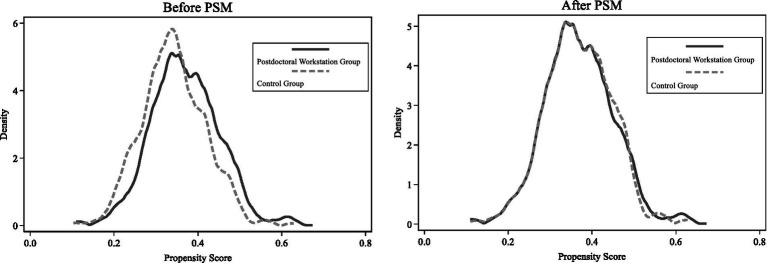
Kernel Density Plot. This figure reports the kernel density function after PSM for the treatment and control groups. Specifically, the probability density functions of the two groups of samples retained after PSM are consistent.

To ensure good comparability of the matched samples, we plotted the kernel density function curves after PSM for the treatment and control groups (see Figure 4). According to the left subfigure, it can be seen that the probability density functions of the propensity score values of the two sample groups are significantly different, and a direct comparison of the differences between these two groups of sample firms without matching is bound to produce a serious estimation bias. The probability density functions of the two groups of samples retained after PSM are consistent (in the right subfigure), which indicates that the characteristics of the two groups of firms are very close after matching, and the selection bias of the samples is corrected.

##### Instrumental variable

To address potential endogeneity issues, we used the natural logarithm of the number of disciplines authorized to offer doctoral degree programs within each province (*Iniv*) as an instrumental variable for two-stage estimation. This variable was selected for a range of reasons: (a) As the number of doctoral programs increases, so does the number of doctoral graduates produce in that area. To facilitate knowledge transfer and mobility and strengthen the management mechanism of doctoral talents, the government administration may increase firms’ requests for postdoctoral workstations to be approved. (b) The Ministry of Education of the People’s Republic of China checks and approves doctoral programs, which is a characteristic that is consistent with the exclusionary constraint of the instrumental variable. The results are reported in Table 6.

##### Entropy balanced

One may argue that firms with postdoctoral workstations are inherently different from those without them and could partially drive our main results. To alleviate this concern, we use entropy balancing to reweight observations in regression models so that treatment and control firms jointly exhibit covariate balance (Hainmueller, 2012). In line with McMullin and Schonberger (2020) in conducting an entropy balanced sample, we match the control firms with all control variables as covariates. Panel A of Table 7 presents the covariate balance results and the differences in covariates between the treatment and control firms. There is no significant difference between the two groups of firms across all covariates used in our primary tests except for the establishment of joint postdoctoral workstations (*t*-stat are close to zero). In Panel B, we re-estimate our main regression models using an entropy balanced sample and still observe a negative and significant coefficient on *Postdoc*, which proves that the baseline results would not suffer from selection bias problems due to observables (Table 7).

**Table 7:** Robustness checks using entropy balanced sample.

Panel A: Covariate balance for entropy balanced sample						
Variables	Treat (*N* = 3,424)		Control (*N* = 6,968)		Difference in means	
	Mean	SD	Mean	SD	Difference	*t*-stat
*Insize*	22.4770	1.1968	22.4766	1.1968	0.00	0.0150
*Lev*	0.4459	0.1898	0.4459	0.1898	0.00	0.0016
*MB*	0.6133	0.2403	0.6133	0.2403	0.00	0.0020
*Cfo*	0.0470	0.0635	0.0470	0.0635	0.00	0.0006
*Age*	16.9888	5.3774	16.9884	5.3774	0.00	0.0032
*Profit*	0.0589	0.1580	0.0589	0.1580	0.00	0.0006
*ROE*	0.0634	0.1348	0.0634	0.1348	0.00	0.0007
*Top1*	0.3119	0.1385	0.3119	0.1385	0.00	−0.0004
*DivDum*	0.8091	0.3931	0.8091	0.3931	0.00	0.0042
*Vol_Nethiring*	0.1166	0.2329	0.1166	0.2329	0.00	0.0001
*Vol_Revt*	0.2013	0.5178	0.2013	0.5178	0.00	−0.0008
*Other_invest*	0.0438	0.0395	0.0438	0.0395	0.00	0.0009
**Panel B: Regression results for postdoctoral workstation and specific types of LIE**						
**Variables**	**Dependent Variable = *|AbnNetHire|***					
	**Overall (1)**	**Over-hiring (2)**	**Under-firing (3)**	**Under-hiring (4)**	**Over-firing (5)**	
*Postdoc*	−0.0199*** (−3.499)	−0.0256 (−1.593)	−0.0566** (−1.998)	−0.0085** (−2.446)	−0.0116* (−1.889)	
*Other Controls*	Yes	Yes	Yes	Yes	Yes	
*Firm and Year*	Yes	Yes	Yes	Yes	Yes	
*Constant*	0.5130*** (6.135)	0.8929*** (3.370)	−0.7146* (−1.666)	0.3771*** (6.903)	0.3804*** (3.219)	
*Adj*. *R^2^*	0.065	0.099	0.294	0.043	0.116	
*N*	10,392	2,363	1,237	5,826	966	

This table reports the covariate balance for the entropy balanced sample (Panel A) and results for Equation (2) using this sample (Panel B). In Panel B, Columns (1) report robustness checks for results in Table 4. Columns (2)–(5) report robustness checks for results in Table 5. Robust standard errors clustered at the firm level to derive t-statistics in parentheses. ***, **, and * represent significance at the 1, 5, and 10% levels, respectively. For brevity, we do not report the coefficients for the control variables.

### Potential mechanism

We have shown that postdoctoral workstation leads to a positive and significant increase in LIE. In this section, we extend the results to shed light on the underlying mechanism of why postdoctoral workstation affects managerial decisions on various aspects of labor investment.

#### Brain gain effect

Previous studies propose that managers can reduce recruitment costs by hiring graduates directly from universities through U-I collaboration (Siegel et al., 2003; Plewa et al., 2014). In the hypothesis development section, we argue that managers are likely to hire more skilled labor in an efficient way to strengthen the complementarity between technological capital and skills and boost postdoctoral workstation accomplishments. At the same time, we may observe accumulation in human capital (brain gain) as skilled workers may decide to move to firms with postdoctoral workstations for a more promising career and higher wages. For unskilled workers, in turn, managers may be inclined to dismiss them to save on human resource costs. Thus, we expect the postdoctoral workstation to be conducive to increasing human capital stock by attracting competent job candidates. Following Cao and Rees (2020) and Wang et al. (2021), we use firms’ actual net hiring (*NetHiring*) in Equation (1), employees with specialist skills (*SkillHiring*), and unskilled production workers (*UnskillHiring*) as the alternative dependent variables in Equation (2) to capture the structure of human capital.

Table 8, Panel A, presents a significant negative association between postdoctoral workstations and firms’ actual net hiring and unskilled production workers. Interestingly, the positive coefficient on *Postdoc* in columns (2) suggests that an increased number of skilled employees are recruited by firms with postdoctoral workstations. The results are consistent with those in Column (2) of Table 5, i.e., postdoctoral workstation primarily mitigates under-firing problem in labor investment, as compared to other types of LIE. In sum, our results suggest that managers are intentionally restructuring the internal workforce to enhance the complementary relationship between postdoctoral researchers and skilled employees.

**Table 8:** The potential mechanism.

Panel A: Mechanism of brain grain effect			
Variables	*NetHiring* (1)	*SkillHiring* (2)	*UnskillHiring* (3)
*Postdoc*	−0.0121^*^ (−1.840)	0.0176^**^ (2.549)	−0.0321^***^ (−3.197)
*Constant*	0.0436 (0.458)	0.0616 (0.700)	0.7277^***^ (5.519)
*Other Controls*	Yes	Yes	Yes
*Firm and Year*	Yes	Yes	Yes
*Adj*. *R^2^*	0.081	0.308	0.256
*N*	10,392	10,392	10,392
**Panel B: Mechanism of knowledge transfer effect**			
**Variables**	***R&D* (1)**	***TrainInvest* (2)**	***Patents* (3)**
*Postdoc*	0.0061*** (3.241)	0.1615* (1.929)	0.2551*** (3.091)
*Constant*	0.0292 (1.202)	1.9444** (2.037)	3.0969*** (2.968)
*Other Controls*	Yes	Yes	Yes
*Firm and Year*	Yes	Yes	Yes
*Adj*. *R^2^*	0.299	0.077	0.063
*N*	10,392	10,392	10,392

This table reports the results for the potential mechanism of the brain grain effect (Panel A) and knowledge transfer effect (Panel B). Robust standard errors clustered at the firm level to derive t-statistics in parentheses. ***, **, and * represent significance at the 1, 5, and 10% levels, respectively. For brevity, we do not report the coefficients for the control variables.

#### Knowledge transfer effect

The knowledge transfer processes in U-I collaboration are a series of complicated activities that undergo an uncertain environmental fluctuation and hence necessitate various forms of arrangements between the relevant firms and universities (Cohen et al., 2002; D’Este et al., 2012; Salimi et al., 2016). In a more recent study, Caloghirou et al. (2021) propose a range of useful strategies for facilitating U-I knowledge transfer, including improving collaborative patenting and R&D information interchange, as well as investing in human resources through joint training programs. For firms’ employees, the above strategies provide the opportunity for “learning by doing” (Rasmussen and Sørheim, 2006), which helps them to develop specialized knowledge and skills (especially for skilled employees), thus leading to a stable labor turnover. Therefore, if knowledge transfer is a potential channel through which postdoctoral workstations affect LIE, we could observe an increasing among the R&D expenditure (*R&D*), training investment (*TrainInvest*), and thus the number of patents (*Patents*). As shown in Panel B of Table 8, the coefficients on *Postdoc* are all positive and significant, suggesting that postdoctoral workstation contributes to managers’ labor investment strategies by strengthening the quality of knowledge transfer processes.

### Moderating effects

#### Moderating effects of national fellows, and political connections

To test hypothesis H3, we construct dummy variables indicating whether the postdoctoral workstations are led by national fellows (*Postdoc_Fellows*) and whether MOST has political connections (*Postdoc_MOST*) by reading the annual report. Specifically, we use text analysis methods and extract keywords such as “fellow,” “fellows of the Chinese Academy of Engineering,” “fellows of the Chinese Academy of Sciences,” “Ministry of Science and Technology,” and “political connection” through the annual report of the firms, and matched them manually with the firm’s announcement, official website and related news reports.

In column (3) of Table 6, although the coefficients are all significantly and negative, as predicted by Hypothesis 3a, firms with political connections to MOST show a more significant improvement in LIE than firms’ postdoctoral workstations without political connections. In contrast, in column (4), postdoctoral workstations led by national researchers had no significant effect on LIE and did not support hypothesis 3b. This can be due to the team’s preference for scholarly above commercial.

#### Moderating effects of university-industry geographic proximity, state ownership, and human capital intensity

In this subsection, we investigate the moderating roles of different characteristics of U-I collaboration by employing several interaction terms.

First, prior literature suggests that geographic proximity facilitates information advantages and knowledge flows, resulting in lower information asymmetry and higher knowledge spillover effect between economic agents (D’Este et al., 2012). To this point, we expect increased knowledge sharing due to the shorter U-I collaboration distance to serve as a complement to the role that the postdoctoral workstation plays in the managerial employment decision. Second, given that government intervention is common and has important implications for a firm’s investment in the Chinese institutional context, the nature of state-owned enterprises (hereafter, SOEs) is to serve as a tool of local government in accomplishing social and political goals (Kong et al., 2018; Luo et al., 2020; Wei et al., 2020). In particular, local governments tend to promote SOEs to create additional employment opportunities in an excess way lest the revelation of regional unemployment pressure leads to negative consequences (Li et al., 2020), even if firms are experiencing a higher-than-expected level of labor investment. In contrast, private firms are less likely vulnerable to political burdens from government intervention (Kong et al., 2018). We thus conjecture that state ownership will attenuate the importance of U-I collaboration in labor. Third, we investigate whether the association is more concentrated in human-capital-intensive firms where employees typically possess higher education and skills and thus are critical for corporate value-enhancing activities. Highly educated or skilled employees can learn from postdoctoral researchers more quickly, and thus transfer innovative ideas into products in an efficient way. As a result, human-capital-intensive firms have strong incentives to adjust their labor force to the optimal level to amplify the achievements of postdoctoral workstations.

To examine the above theoretical predictions, we use the average distance of a firm’s headquarters from all Project 211 universities in its province (*Distance*) as an inverse measure for U-I geographic proximity. To proxy for state ownership, we identify whether a firm is SOE based on the type of the ultimate controlling shareholders. Following Cao and Rees (2020), we use the proportion of employees with a Bachelor’s degree or above and professional skills to capture the human capital intensity (denoted by *Education_intensity* and *Skill_intensity*, respectively). We then test the moderating effects by augmenting Equation (2) to include interaction terms.

The results in Table 9 show that the key variables of interest—the interaction terms between the indicator of postdoctoral workstation and the moderating variables—are statistically significant. In particular, the coefficient on Postdoc*Distance is positive, indicating that geographic proximity complements postdoctoral workstations in lowering inefficient labor investments. Such a finding is consistent with the view that U-I collaboration facilitates LIE through the information and knowledge transfer channel. Moreover, the favorable impact of postdoctoral workstations on LIE is more pronounced for non-SOEs, while SOEs have higher labor adjustment costs due to the political burden of maintaining local employment (Kong et al., 2018). Finally, the coefficients of both variables Postdoc*Education_intensity and Postdoc*Skill_intensity are negatively significant, which are consistent with the view that managers in human-capital-intensive firms that rely on knowledge-based operations are more likely to cater to the interests of postdoctoral researchers, and hence have a stronger incentive to adjust and to invest efficiently in labor.

**Table 9:** The moderating role of university-industry collaboration characteristics.

Variables	Dependent Variable = *|AbnNetHire|*			
	(1)	(2)	(3)	(4)
Postdoc*Distance	0.1113** (2.690)			
Postdoc*SOE		0.0089** (2.135)		
Postdoc*Education_intensity			−0.0779*** (−2.647)	
Postdoc*Skill_intensity				−0.0995** (−2.759)
*Distance*	−0.4275*** (−7.493)			
*SOE*		−0.0259*** (−4.961)		
*Education_intensity*			0.0309* (1.668)	
*Skill_intensity*				0.0082 (0.203)
*Postdoc*	−0.0596*** (−3.461)	−0.0222*** (−3.853)	0.0003** (2.034)	0.0019** (2.218)
*Constant*	0.5391*** (6.074)	0.4034*** (4.832)	0.4486*** (5.716)	0.4447*** (4.947)
*Other Controls*	Yes	Yes	Yes	Yes
*Firm and Year*	Yes	Yes	Yes	Yes
*Adj*. *R^2^*	0.083	0.064	0.067	0.066
*N*	10,392	10,392	10,392	10,392

This table reports the results for the moderating effects of university-industry geographic proximity, state ownership, as well as human capital intensity on the association between postdoctoral workstation and labor investment efficiency. Robust standard errors clustered at the firm level to derive t-statistics in parentheses. ***, **, and * represent significance at the 1, 5, and 10% levels, respectively. For brevity, we do not report the coefficients for the control variables.

### What do firms gain from increasing LIE?

In line with the literature on U-I collaboration, we focus on the economic consequences of postdoctoral workstations on firms’ value-enhancing activities. As prior studies find that the resolution of labor investment inefficiency improves a firm’s productivity and value (e.g., Jung et al., 2014; Cao and Rees, 2020), we thus explore whether firms are gaining in terms of productivity and firm value. Following Wang et al. (2021), we conduct a first-order difference model, whereby the variables for economic consequences and LIE are in change forms under the condition of the constant postdoctoral workstation. We use the total factor productivity (*TFP*) calculated by the OP method and Tobin’s Q (*Tobinq*) to proxy for productivity and firm value, respectively. Table 10 shows that the coefficients of interaction terms between the *Postdoc* and the change in labor investment inefficiency are positively significant. These results indicate that firms with postdoctoral workstations will achieve higher performance growth when adjusting labor investment toward optimal levels.

**Table 10:** Postdoctoral workstation, LIE, and performance growth.

Variables	Dependent Variable = *performance growth*	
	*ΔTFP* (1)	*ΔTobinq* (2)
*Postdoc*	−0.0049 (−0.945)	0.0120 (0.966)
*Δ|AbnNetHire|*	0.0477** (2.407)	−0.1531*** (−4.621)
Postdoc*Δ|AbnNetHire|	0.0822** (2.271)	0.0892* (1.683)
*Constant*	0.4617^***^ (5.362)	−0.1291 (−0.584)
*Other Controls*	Yes	Yes
*Firm and Year*	Yes	Yes
*Adj*. *R^2^*	0.080	0.304
*N*	8,033	8,589

This table reports the economic consequences by investigating how changes in LIE affect the firms’ performance growth in the presence of postdoctoral workstation. Robust standard errors clustered at the firm level to derive t-statistics in parentheses. ***, **, and * represent significance at the 1, 5, and 10% levels, respectively. For brevity, we do not report the coefficients for the control variables.

## Conclusion

Despite the importance of U-I collaboration for firms’ value creation, academic studies analyzing its potential impact on managers’ various investment decisions are scant. In this paper, we utilize the staggering establishment of joint postdoctoral workstations in firms as a quasi-natural experiment relative to U-I collaboration and discover that the postdoctoral workstation facilitates more efficient investment in human capital, and the higher the operational quality of the workstation, the greater significant the increase in LIE. Furthermore, we find that postdoctoral workstations aid in the reduction of over-investment (under-firing) and under-investment (under-hiring and over-firing) among the various types of labor investment inefficiencies. We confirm the validity of this finding by conducting the event study approach, placebo test, propensity score matching, instrumental variable, and entropy balancing. Further analysis demonstrates the underlying channel through which brain gain effect, notably the workforce restructuring for skilled labor, and knowledge transfer effect drive our primary results. We also present evidence suggesting that the benefit of postdoctoral workstations is more pronounced for firms located closer to prestigious universities, human-capital-intensive firms, have political connections, and without national fellows’ lead. While the government intervention through state ownership slightly decreases this favorable impact. Finally, we find that firms with postdoctoral workstations have higher productivity and firm value growth when improving labor investment efficiency. Overall, this strand of evidence supports that postdoctoral workstations serve as a key vessel of knowledge transfer, as well as enrich managerial learning about human resource development, and thus enhance firms’ labor investment efficiency.

Our findings have several implications for how firms develop human resources. First, knowledge as a non-material resource is a process that enhances organizational capacity (Gasik, 2011), against the backdrop of China’s demographic dividend gradually fading, the establishment of postdoctoral workstations can stimulate the upgrading of human capital and contribute the driving force to firms’ value-enhancing activities. Therefore, managers must seek out U-I collaboration, as well as access to more high-end talents. HUAWEI, for example, has established postdoctoral workstations with prestigious universities in Beijing and Shanghai to boost both the quantity and quality of postdoctoral training projects. This approach echoes the view of Bastos et al. (2021) that firms acquire new knowledge from collaboration, therefore increasing the possibilities for innovation and economic performance. Second, for the human resource development strategies, we believe that postdoctoral researchers should be incentivized differently from other employees. On the one hand, for postdocs, managers need to encourage them to gain sufficient practical experience by entering front-line production units alongside their R&D activities. On the other hand, as Salimi et al. (2016) and Caloghirou et al. (2021) point out, managers could spend more budget on on-the-job training programs for other employees. The complementarity between postdoctoral workstations and skilled employees would be enhanced by taking this strategy.

We acknowledge several potential limitations of this study. First, due to data availability, our joint postdoctoral program data can only be combined with A-share listed firms, which typically have larger workforce sizes. This may pose a threat to the generalizability of the findings, as start-ups and small firms face lower labor adjustment costs than larger firms. Second, it should be noted that we only focus on the role of efficient labor investments in promoting productivity and firm value based on human resource management theory. Future studies, thus, could investigate the other economic consequences of postdoctoral workstations under the framework of principal-agent theory and signaling theory. Third, and research outlook, is to further extend our findings to all types of projects and temporary highly skilled employees. We will also make more efforts to think about hiring professional service providers in a shorter time and what the practical impact of project managers is in terms of knowledge transfer.

## Data availability statement

The raw data supporting the conclusions of this article will be made available by the authors, without undue reservation.

## Author contributions

GH and KY contributed to conception and design of the study and organized the database. YL performed the statistical analysis and wrote the first draft of the manuscript. All authors wrote sections of the manuscript, contributed to manuscript revision, read, and approved the submitted version.

## Funding

This study was funded by University of International Business and Economics Graduate Research Innovation Project (Grant no. 202259).

## Conflict of interest

The authors declare that the research was conducted in the absence of any commercial or financial relationships that could be construed as a potential conflict of interest.

## Publisher’s note

All claims expressed in this article are solely those of the authors and do not necessarily represent those of their affiliated organizations, or those of the publisher, the editors and the reviewers. Any product that may be evaluated in this article, or claim that may be made by its manufacturer, is not guaranteed or endorsed by the publisher.

## Footnotes

^1^ http://www.gov.cn/zhengce/content/2015-12/03/content_10380.htm
